# Moderate glucose control results in less negative nitrogen balances in medical intensive care unit patients: a randomized, controlled study

**DOI:** 10.1186/cc11299

**Published:** 2012-04-05

**Authors:** Chien-Wei Hsu, Shu-Fen Sun, Shoa-Lin Lin, Hsiu-Hua Huang, Kam-Fai Wong

**Affiliations:** 1 Intensive Care Unit, Department of Medicine, Kaohsiung Veterans General Hospital, 386 Ta-Chung 1st Road, Kaohsiung City 813, Taiwan; 2 Medicine Department, School of Medicine, National Yang-Ming University, 155 Sec. 2 Linong street, Taipei City 112, Taiwan; 3 Department of Physical Medicine and Rehabilitation, Kaohsiung Veterans General Hospital, 386 Ta-Chung 1st Road, Kaohsiung City 813, Taiwan; 4 Department of Food and Nutrition, Taipei Veterans General Hospital, 201 Sec. 2 Shipai Road, Beitou District, Taipei City 112, Taiwan; 5 Institute of Statistics, National University of Kaohsiung, 700 Kaohsiung University Road, Nanzih District, Kaohsiung City 811, Taiwan

## Abstract

**Introduction:**

Hyperglycemia and protein loss are common in critically ill patients. Insulin can be used to lower blood glucose and inhibit proteolysis. The impact of moderate insulin therapy on protein metabolism in critically ill patients has not been evaluated. We compared urinary nitrogen excretion, nitrogen balance, serum albumin concentrations, prealbumin concentrations, and clinical outcomes between patients receiving moderate insulin therapy (MIT) and conventional insulin therapy (CIT) in a medical ICU.

**Methods:**

Patients were randomly divided into groups and treated with MIT (glucose target 120 to 140 mg/dl) or CIT (glucose target 180 to 200 mg/dl). Calories and protein intake were recorded each day. On days 3, 7 and 14, the 24-hour urinary nitrogen excretion, nitrogen balance, and serum albumin and prealbumin concentrations were measured. Clinical outcomes data were collected.

**Results:**

A total of 112 medical ICU patients were included, with 55 patients randomized to the MIT group and 57 patients randomized to the CIT group. Patients treated with MIT showed a trend towards increased nitrogen balance (*P *= 0.070), significantly lower urinary nitrogen excretion (*P *= 0.027), and higher serum albumin (*P *= 0.047) and prealbumin (*P *= 0.001) concentrations than patients treated with CIT. The differences between the two groups were most significant on day 3, when all factors showed significant differences (*P *< 0.05).

**Conclusions:**

Moderate glucose control results in less negative nitrogen balances in medical ICU patients. Differences are more significant in the early stages compared with the late stages of critical illness.

**Trial registration:**

ClinicalTrial.Gov NCT 01227148

## Introduction

Hyperglycemia, which is common in critically ill patients, occurs even in those patients who have not previously had diabetes [1,2]. Critical illness is associated with increased circulating concentrations of proinflammatory cytokines, such as TNFα, IL-1, and IL-6, which may be important mediators of insulin resistance and hyperglycemia [3]. Altered glucose metabolism results from the release of counter-regulatory hormones. Epinephrine and cortisol oppose the normal action of insulin, leading to increased adipose tissue lipolysis and skeletal muscle proteolysis [4]. Reports state that pronounced hyperglycemia may lead to complications or a poor clinical outcome in such patients [5,6]. The maintenance of normoglycemia using insulin therapy has been shown to significantly reduce morbidity and mortality in critically ill patients [5,7,8]. However, some studies failed to demonstrate improved outcome [9-12], and even a higher mortality rate was found [13] Intensive insulin therapy has been associated with a significantly higher risk of hypoglycemia [5,7], resulting in concern regarding the safety of intensive insulin therapy. Glycemic control to a moderately tight range is not inferior to euglycemia and is clearly safer in critically ill patients [14]. Patients with glucose levels of 130 mg/dl were reported to have a significantly lower incidence of infection and sepsis and a lower mortality rate [15].

Protein loss, which is also common in critically ill patients, is thought to arise from both increased proteolysis [16] and diminished protein synthesis [17]. Protein wasting can negatively affect patient outcome [18]. The use of insulin to promote an anabolic response during critical illness is a promising therapeutic approach. Animal studies have shown that strict insulin therapy normalizes organ nitrogen contents in diabetic rats [19]. Woolfson and colleagues demonstrated that insulin has important protein-sparing effects in severely ill trauma patients [20]. Shiozaki and colleagues showed that burn patients who received higher doses of insulin maintained a better nitrogen balance than a lower-dose group [21].

The purpose of this study was to investigate the differences in urinary nitrogen excretion, nitrogen balance, serum albumin and prealbumin concentrations, and clinical outcomes between moderate insulin therapy (MIT) and conventional insulin therapy (CIT) in critically ill patients.

## Materials and methods

### Study design

This study was a prospective, randomized, controlled trial conducted in an adult medical ICU of a tertiary medical center that has 1,235 beds in total, 59 of which are adult ICU beds. Trial and consent forms were approved by the Institutional Review Board of Kaohsiung Veterans General Hospital. The study was carried out between January 2006 and December 2006. Procedures were in accordance with the Helsinki Declaration.

### Subjects

**Figure 1:**
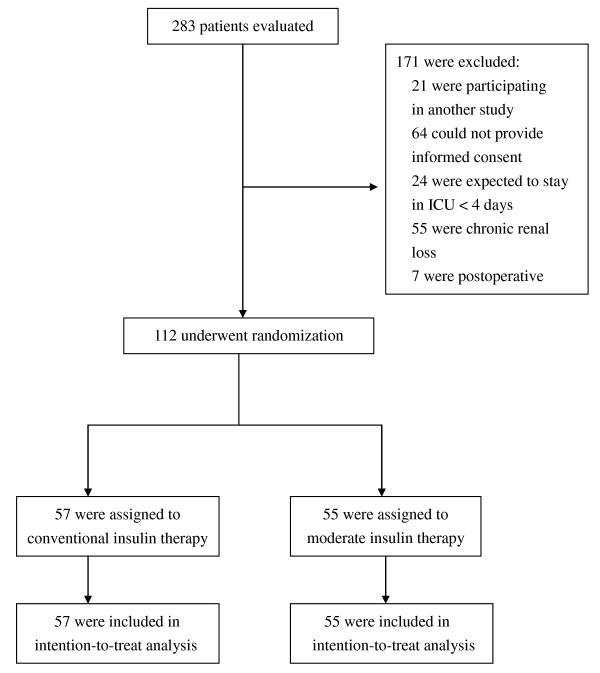
**Assessment, randomization, and follow-up of the study patients**. For detailed characteristics of randomized patients, see Table 1.

Patients aged 18 or over who were admitted to the medical ICU with blood glucose concentrations over 180 mg/dl were eligible for inclusion. The criteria for exclusion included prior surgical treatment, pregnancy, participation in another study, patients with chronic renal loss, and patients who were expected to require treatment in the ICU for less than 4 days. Chronic renal loss was defined as persistent acute renal failure with the complete loss of kidney function for more than 4 weeks [22]. The first author (C-WH) enrolled participants. A total of 283 patients were evaluated and 171 patients were excluded. The remaining 112 patients were eligible for the study (Figure 1).

### Insulin therapy

After informed consent was obtained from patients or the next of kin, patients were randomly assigned to receive either MIT or CIT using software-generated simple randomization procedures (computerized random number) without blocking. Except for the interventionists (nurses and doctors), patients and other staff members were not informed of the group assignments. In the MIT group, continuous insulin infusion was started when the blood glucose concentrations exceeded 140 mg/dl in order to maintain a blood glucose concentration between 120 and 140 mg/dl. The insulin dose was adjusted using a neuro-fuzzy method; the insulin protocol is shown in Additional file 1. In the CIT group, continuous insulin infusion was delivered when blood glucose concentrations exceeded 200 mg/dl; the insulin dose was then adjusted to maintain a blood glucose concentration between 180 and 200 mg/dl.

All patients were fed either intravenously (total parenteral nutrition) or enterally starting the day after ICU admission. Enteral feeding was attempted as early as possible at the discretion of the attending physician. Once enteral feeding was started, patients were administered full-strength isotonic commercial formula Jevity (Abbott Laboratories, Brockville, ON, Canada) commencing at 20 ml/hour, and increased by 20 ml/hour every 4 hours to satisfy energy and protein requirements recommended by the clinical dietitian following the Canadian clinical practice guidelines for critically ill patients [23]. All patients did not receive an albumin infusion during the study period.

### Data collection

At the time of randomization, demographic data and clinical characteristics were obtained. Blood glucose concentrations were measured upon admission and, subsequently, every 1 to 4 hours in all patients. Baseline serum albumin concentrations, prealbumin concentrations (Hitachi 7600; Hitachi, Tokyo, Japan), serum creatinine, and 24-hour urinary urea nitrogen (UUN) levels were collected and evaluated after beginning the study, and these data were collected and evaluated on days 3, 7, and 14 of the study. Additionally, we used full 24-hour urine collection rather than spot urine samples to determine the UUN. Blood cultures were obtained whenever the central body temperature exceeded 38.5°C or other clinical signs of sepsis were present. Blood transfusions were conducted according to the protocol when hemoglobin levels decreased to below 7 g/dl and were continued until target hemoglobin levels of 7 to 9 g/dl were attained. A higher hemoglobin level was required in patients with special circumstances; for example, myocardial ischemia, severe hypoxemia, acute hemorrhage, or lactic acidosis [24].

From the time of randomization to the time of discharge from the ICU, daily caloric and protein intakes, all blood glucose measurements, doses of insulin, red-blood-cell transfusions, blood cultures that were positive for pathogenic organisms, renal function, gastrointestinal bleeding, moderate hypoglycemia, and severe hypoglycemia were recorded.

### Definitions

Patients were classified as having diabetes on the basis of their medical history. Previous treatment with corticosteroids was defined as treatment with systemic corticosteroids for 72 hours or more immediately before randomization. Sepsis was defined by the presence of both infection and a systemic inflammatory response [25]. Acute renal injury was defined when serum creatinine concentrations increased by 2.0 times or the glomerular filtration rate decreased by more than 50% [22]. Creatinine clearance was calculated using the Cockcroft-Gault equation [26]:

$$\left(\text{14}0-\text{age}\left(\text{years}\right)\right)\times \text{body}\;\text{weight}\left(\text{kg}\right)\times 0.\text{85}\left(\text{if}\;\text{female}\right)/\text{72}\times \text{serum}\;\text{creatinine}\left(\text{mg}/\text{dl}\right)$$

Bloodstream infection was diagnosed either when a recognized pathogen was isolated from a blood culture, or in the presence of one of fever (> 38°C), chills, or hypotension and any of the following: a common skin contaminant isolated from two blood cultures that were drawn on separate occasions; a common skin contaminant isolated from the blood culture of a patient with an intravascular access device, and the physician instituted appropriate antimicrobial therapy; or a positive antigen blood test [27].

Gastrointestinal bleeding was defined as the presence of hematemesis, melena, bright red blood per rectum, or a coffee grounds-like substance that was aspirated from the feeding tube. Patients with hypoglycemia were defined as those who developed at least one episode of hypoglycemia. A moderate hypoglycemic event was defined as a blood glucose concentrations ≤ 60 and > 40 mg/dl. A severe hypoglycemic event was defined as blood glucose concentration ≤ 40 mg/dl.

The 24-hour nitrogen balance was calculated using the following formula [28]:

$$\text{Nitrogen}\;\text{balance}=\left(\text{protein}\;\text{intake}/\text{6}.\text{25}\right)-\left(\text{UUN}+\text{4}\right)$$

The difference in daily insulin dose between the two groups was the total mean insulin dose of the MIT group minus the total mean insulin dose of the CIT group on the same day.

### Outcomes

Primary outcomes included 24-hour UUN levels, nitrogen balance, serum albumin concentrations, and prealbumin concentrations. Secondary outcomes included ICU days, ventilator days, hospital days, acute renal injury, bloodstream infection, blood transfusions, gastrointestinal bleeding, moderate hypoglycemia, severe hypoglycemia, and hospital mortality rate.

### Statistical analysis

All data were analyzed by SPSS version 12.0 (SPSS, Inc., Chicago, IL, USA). Data are presented as the mean ± standard deviation, median (interquartile range), or number (percentage). All data were analyzed according to the intention-to-treat principle. To detect differences of nitrogen balance using a two-sided 5% significance level and a power of 85% when the difference and standard deviation were equal to 0.80 and 1.97, a sample size of 55 patients per group was necessary. This value was based on previous studies involving nitrogen balance and different doses of insulin treatment in a burn ICU [21]. Primary outcomes, such as 24-hour UUN levels, nitrogen balance, serum albumin concentrations, and prealbumin concentrations were analyzed with a generalized linear model for repeated measures using dummy variables. Student's *t *test was used to compare continuous variables with normally distributed data. Wilcoxon's rank-sum tests were used to compare continuous variables with non-normally distributed data. Chi-square tests were used to compare dichotomous variables. All *P *values were two-tailed. *P *< 0.05 was considered significant.

## Results

### Patient characteristics

**Figure 2:**
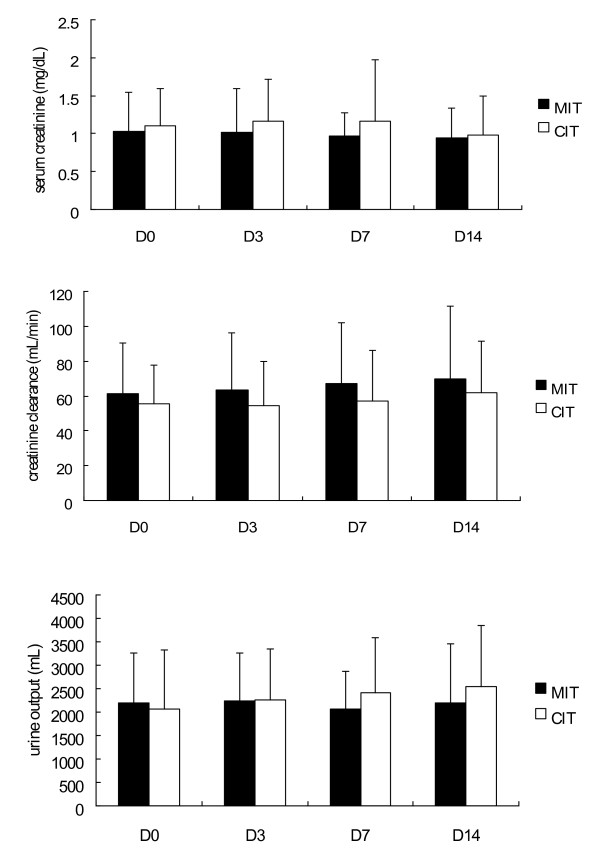
**Serum creatinine, creatinine clearance and 24-hour urine output on days 0, 3, 7, and 14**. Top, serum creatinine; middle, creatinine clearance; bottom, 24-hour urine output. Filled bars, patients receiving moderate insulin therapy (MIT group); open bars, patients receiving conventional insulin therapy (CIT group).

All participants were recruited from April 2006 to December 2006. This trial ended when it reach the sample size goal. Of the 112 patients randomized to the study, 55 patients received MIT and 57 patients received CIT. All patients were available for the intention-to-treat analysis (Figure 1). Table 1 shows the patient baseline characteristics of all the patients enrolled in the study. Demographic data at the time of randomization showed no significant differences in any of the parameters. Figure 2 shows serum creatinine, creatinine clearance, and urine output during the study period between the two groups; there were no significant differences. The two groups shared similar background characteristics.

**Table 1:** Demographic data for all patients

Characteristic	MIT group (*n *= 55)	CIT group (*n *= 57)	*P *value
Primary ICU admitting diagnosis			
Sepsis	25	26	
Respiratory	11	12	
Neurologic	9	7	
Cardiovascular	3	5	
Gastrointestinal or liver	2	3	
Hematologic or oncologic	3	1	
Renal	1	2	
Metabolic	1	1	
Age	68.1 ± 16.3	70.4 ± 12.1	0.40
Body weight (kg)	61.4 ± 9.6	62.5 ± 9.8	0.57
Gender (female/male)	16/39 (41%)	16/41 (39%)	0.90
Body mass index (kg/m^2^)	23.6 ± 3.0	23.9 ± 3.8	0.72
History of diabetes	19/55 (34.5%)	22/57 (38.6%)	0.67
Previous corticosteroid treatment	14/55 (25.4%)	16/57 (28.1%)	0.77
Use of inotropes	15/55 (27.3)	12/57 (21.1)	0.44
APACHE II score	20 (17 to 25)	21 (16.5 to 26.5)	0.94
Patients with total parenteral nutrition	2/55 (3.6%)	2/57 (3.5%)	0.97
Intake of nutrients			
Carbohydrate (%)	54.3 ± 0.2	54.4 ± 0.2	0.84
Protein (%)	16.4 ± 0.1	16.4 ± 0.2	0.50
Fat (%)	29.3 ± 0.2	29.2 ± 0.8	0.55
At randomization			
Blood glucose (mg/dl)	229.2 ± 39.3	231.1 ± 52.1	0.93
Serum hemoglobin (g/dl)	10.9 ± 2.2	10.8 ± 2.3	0.80
Serum ALT (U/l)	59.1 ± 79.3	63.8 ± 83.0	0.76
Serum total bilirubin (mg/dl)	1.1 ± 0.4	1.2 ± 0.4	0.88
UUN (g/BSA/24 hours)	7.0 ± 3.7	6.7 ± 3.8	0.78
Nitrogen balance (g/BSA/day)	-4.8 ± 5.9	-4.7 ± 4.5	0.93
Serum albumin (mg/dl)	1,949.9 ± 376.0	1,955.4 ± 506.5	0.95
Serum prealbumin (mg/dl)	11.3 ± 5.2	11.2 ± 4.5	0.87

Data presented as *n*, mean ± standard deviation, or median (interquartile range). ALT, alanine aminotransferase; APACHE, Acute Physiology and Chronic Health Evaluation; BSA, body surface area; CIT, conventional insulin therapy; MIT, moderate insulin therapy; UUN, urinary urea nitrogen.

### Nutrition and blood glucose control

**Figure 3:**
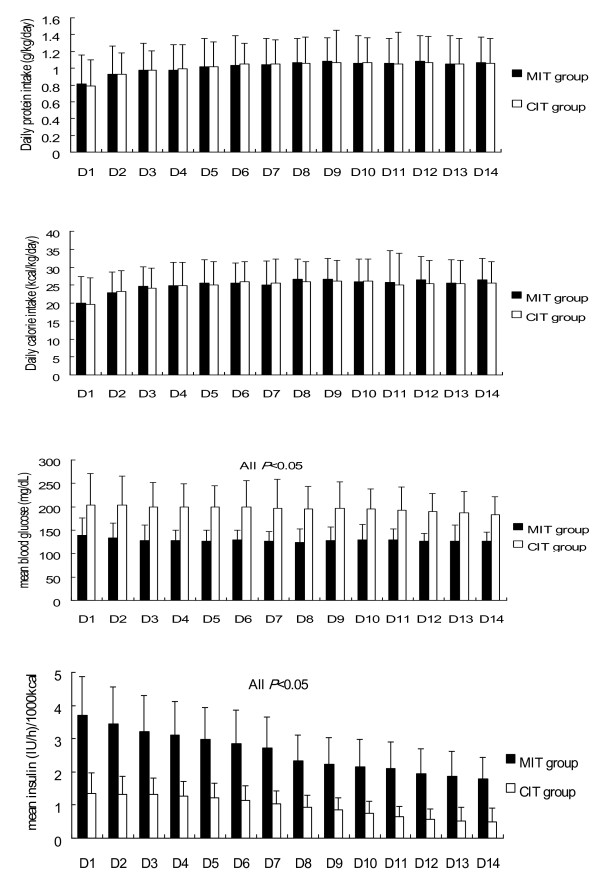
**Daily protein, calories intake, mean blood glucose levels and insulin dose**. Daily protein intake (top), daily caloric intake (second from top), mean blood glucose levels (second from bottom), and insulin dose (bottom) during the 2-week study period in the medical ICU. Filled bars, moderate insulin therapy (MIT) group; open bars, conventional insulin therapy (CIT) group.

**Figure 4:**
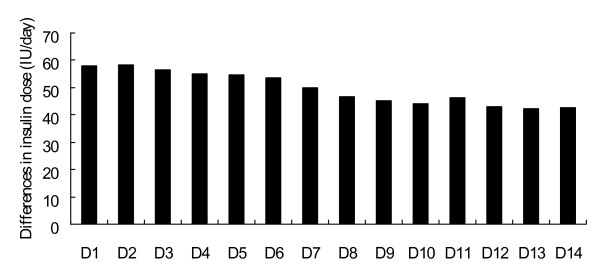
**Differences in mean daily insulin doses between moderate and conventional insulin therapy**. Differences in mean daily insulin doses between the moderate insulin therapy group and the conventional insulin therapy group.

Actual daily protein (grams per day and per kilogram of body weight) and caloric (calories per day and per kilogram of body weight) intake, blood glucose concentrations, and insulin dose (per hour corrected for caloric intake) are shown in Figure 3. Daily protein and caloric intake progressively increased until day 5, but then fluctuated after day 5 in both groups. The two treatment groups had similar protein and caloric intakes. The mean blood glucose concentration was 125.3 ± 2.4 mg/dl in the patients of the MIT group and 199.9 ± 4.0 mg/dl (*P *< 0.01) in the patients of the CIT group. The median daily insulin dose was 82 (69 to 111) units/day in the patients of the MIT group and 37 (26 to 43) units/day (*P *< 0.01) in the patients of the CIT group (Table 2). To maintain the low blood glucose concentrations, patients in the MIT group were administered a significantly higher insulin dose than patients in the CIT group. The insulin requirement per calorie decreased each day in both groups (Figure 3). The difference in mean daily insulin doses between the two groups ranged from 58 IU/day on day 1 to 43 IU/day on day 14. The difference in insulin requirements per day between the two groups decreased each day (Figure 4).

**Table 2:** Clinical outcomes

Outcome variable	MIT group (*n *= 55)	CIT group (*n *= 57)	*P *value
Mean blood glucose (mg/dl)	125.3 ± 17.8	199.9 ± 30.2	< 0.01
Daily dose of insulin (unit/day)	82 (69 to 111)	37 (26 to 44)	< 0.01
ICU days	14 (9 to 19)	15 (11 to 26)	0.26
Ventilator days	20 (11 to 30)	23 (11 to 43.5)	0.19
Hospital days	27 (15 to 36)	32 (18.5 to 51.3)	0.052
Acute renal injury	3/55 (5.5%)	7/57 (12.3%)	0.10
Bloodstream infection	1/55 (1.8%)	3/57 (5.3%)	0.33
Red-blood-cell blood transfusion	12/55 (21.8%)	15/57 (26.3%)	0.74
Gastrointestinal bleeding	7/55 (12.7%)	7/57 (12.3%)	0.83
Number of mild hypoglycemia	10/55 (18.2%)	6/57 (10.5%)	0.37
Rate of moderate hypoglycemia per 100 treatment days	2.4	1.5	0.18
Number of severe hypoglycemia	2/55 (3.6%)	1/57 (1.8%)	0.53
Rate of severe hypoglycemia per 100 treatment days	0.3	0.2	0.44
Hospital mortality rate	18/55 (32.7%)	28/57 (49.1%)	0.08

Data presented as *n*, mean ± standard deviation, or median (interquartile range). CIT, conventional insulin therapy; MIT, moderate insulin therapy.

### Primary outcomes

**Figure 5:**
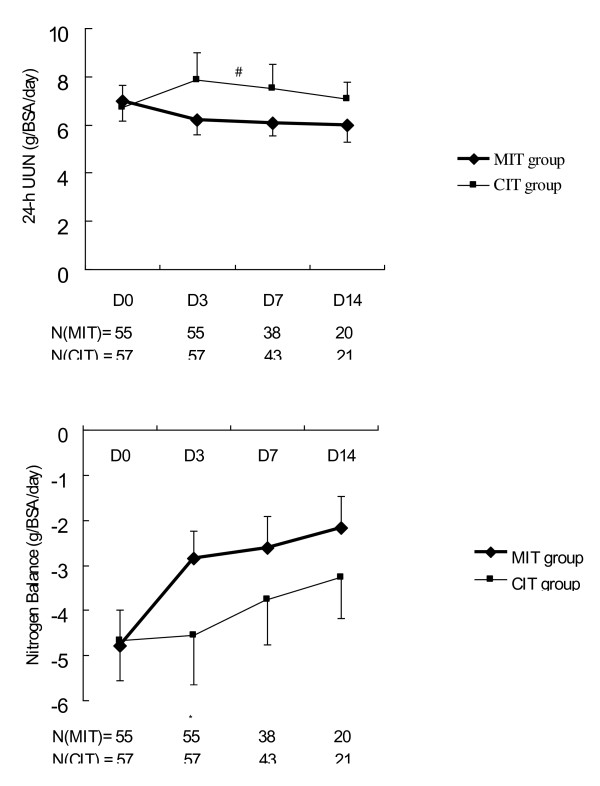
**Twenty-four-hour urinary urea nitrogen and nitrogen balance in patients receiving moderate or conventional insulin therapy**. Top, 24-hour urinary urea nitrogen (UUN); bottom, nitrogen balance. Data represent the mean ± standard deviation. A generalized linear model of repeated measurements showed statistically significant differences between the two groups: ^#^*P *= 0.027 for entire study period, *P < 0.05 for day 3. BSA, body surface area; CIT, conventional insulin therapy; MIT, moderate insulin therapy.

**Figure 6:**
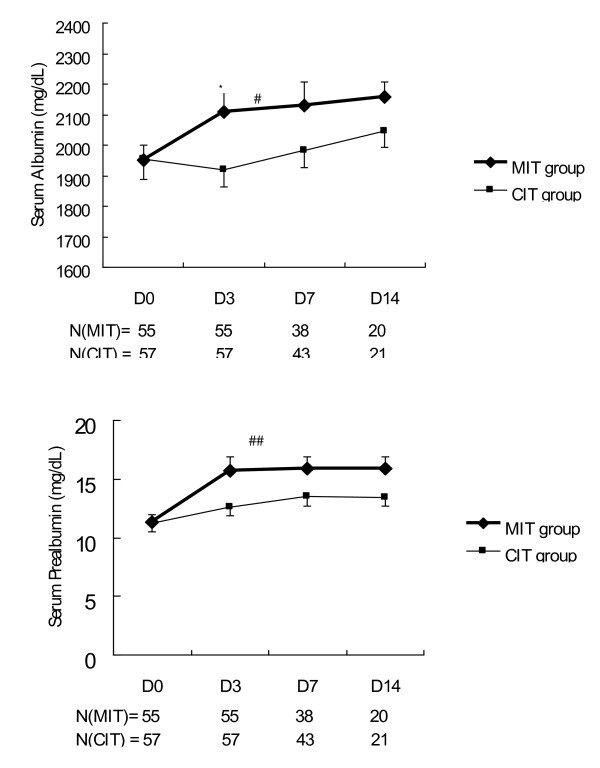
**Serum albumin and prealbumin levels in patients receiving moderate or conventional insulin therapy**. Top, serum albumin; bottom, serum prealbumin. Data represent the mean ± standard deviation. A generalized linear model of repeated measurements showed statistically significant differences: ^#^*P *= 0.047 for entire study period, ^##^*P *= 0.001 for entire study period, *P < 0.05 for day 3. CIT, conventional insulin therapy; MIT, moderate insulin therapy.

Both groups showed a trend of decreasing levels of excreted urinary nitrogen, better nitrogen balance, and increasing serum albumin and prealbumin concentrations during their hospital courses. A generalized linear model for repeated measurements using dummy variables revealed that there were significantly lower 24-hour urine nitrogen excretion (*P *= 0.027) (Figure 5) and higher serum albumin (*P *= 0.047) and prealbumin (*P *= 0.001) concentrations (Figure 6) during the study period in the MIT group. The MIT group exhibited a higher nitrogen balance than the CIT group; however, this difference was not statistically significant (*P *= 0.070). Differences in 24-hour urine nitrogen excretion, nitrogen balance, serum albumin concentrations, and prealbumin concentrations between the two groups were most significant on day 3 when all factors showed significant differences (*P *< 0.05). These differences decreased after day 3.

### Secondary outcomes

There were no statistically significant differences in ICU days, ventilator days, hospital days, rate of acute renal injury, bloodstream infection, red-blood-cell transfusion, and gastrointestinal bleeding in the two groups (Table 2).

Moderate hypoglycemia (blood glucose levels ≤ 60 mg/dl and > 40 mg/dl) occurred more often in the MIT group than in the CIT group, but this difference was not statistically significant (18.2% vs. 10.5%, *P *= 0.10; 2.4 episodes vs. 1.5 episodes per 100 treatment days, *P *= 0.18) (Table 2). The CIT group also exhibited a lower rate of severe hypoglycemia (blood glucose levels ≤ 40 mg/dl), but the difference was not significant (3.6% vs. 1.8%, *P *= 0.53; 0.3 episodes vs. 0.2 episodes per 100 treatment days, *P *= 0.44). No hemodynamic deterioration, convulsions, or neurological sequelae were noted in association with any hypoglycemic event in either group.

Patients with MIT had a trend for lower hospital mortality rate than that of patients with CIT, but they did not reach a statistically significant difference.

## Discussion

The results of this study showed that MIT could significantly improve nitrogen balance in the early stages of critical illness. Patients treated with MIT had significantly lower urinary nitrogen excretion and higher serum albumin and prealbumin concentrations than patients treated with CIT.

In the present study, both groups showed a negative nitrogen balance during the study period. Although the nitrogen balance was negative, both groups showed improvement during the study period. Protein is rapidly broken down in critically ill patients who are subjected to high stress [18]. Hypermetabolism in patients under stress is associated with an increased negative nitrogen balance, and a positive nitrogen balance is difficult to attain in hypermetabolic patients [29]. Equilibrium between energy intake and energy expenditure, early feeding, and a high-protein diet may improve nitrogen balance [30-32]. In this study, nitrogen balance was improved using moderate glucose control. This improvement may have arisen from higher dose insulin administered to the MIT group during the hospital course. Higher infused doses of insulin can result in a marked reduction in UUN excretion and a better nitrogen balance [21].

Differences in nitrogen balance between the two treatment groups were significant in the early stages of critical illness, but decreased over time. This observation may be related to the insulin dose. Differences in insulin dose between the two groups were more significant in the early stages than in the late stages.

The effect of insulin on protein metabolism appears primarily to be due to inhibition of proteolysis [33-35], although increased protein synthesis has been reported [36]. In healthy subjects, insulin inhibits proteolysis in a dose-dependent manner [37]. Insulin binding to its receptors activates the insulin receptor substrate pathway, leading to activation of protein kinase; protein kinase B modulates enzyme activities that affect nitric oxide generation and control protein metabolism [38].

Insulin also has an anabolic effect that is beneficial to critically ill patients; it may increase levels of insulin-like growth factor (IGF)-1, a mediator of anabolic growth hormone action, and decrease hepatic synthesis of IGF-1 binding protein, leading to the increased bioavailability of IGF-1 [39]. In a study involving rats, IGF-1 administration significantly improved nitrogen balance [40].

Critical illness is characterized by hypermetabolism and catabolism, leading to peripheral protein waste [41,42]. Proinflammatory mediators enhance catabolism and hypermetabolism by inhibition of the growth hormone-IGF-I-insulin axis [43-45]. Insulin improves hypermetabolism by affecting proinflammatory cytokine production and hepatic signal transcription factor expression [46]; it attenuates the inflammatory response by decreasing the proinflammatory cascade and increasing the anti-inflammatory cascade. By decreasing proinflammatory mediators, liver constitutive proteins such as albumin and prealbumin are increased [47]. Inflammation reduces the albumin concentration by decreasing its rate of synthesis and increasing transfer of albumin out of the vascular compartment [48].

Although our data and previous studies have shown that insulin benefits protein metabolism, the extent to which the benefit is derived from the correction of hyperglycemia as opposed to the direct effects of insulin remains unclear [49]. Van den Berghe and colleagues revealed that the lowering of blood glucose levels rather than the amount of infused insulin is related to the effects of insulin therapy on morbidity [7]. Acute hyperglycemia enhances proteolysis in the entire body during hyperinsulinemia in normal men [50]. Additionally, Whyte and colleagues demonstrated that the administration of insulin which resulted in supraphysiological concentrations did not attenuate protein breakdown and had no effect on the net protein balance in critically ill patients [51].

For patients receiving total parenteral nutrition, glucose and lipid ratios influence the nitrogen balance. A previous study showed that total parenteral nutrition at a glucose/lipid ratio of 50/50 induced a significantly higher nitrogen balance than an 80/20 ratio with an isocaloric isonitrogenous parenteral nutrition formula [52]. In our study, few patients received total parenteral nutrition and they had a similar glucose/lipid ratio; thus, the glucose/lipid ratio is not a confounding factor for maintaining nitrogen balance.

A meta-analysis study showed that intensive insulin therapy has no advantage in reducing the mortality rate, but significantly increases the risk of hypoglycemia [53]. Our study demonstrated that most patients with hypoglycemia have a moderate form; severe hypoglycemia was rarely observed. Target glucose levels in our study were 120 to 140 mg/dl, which was higher than those in intensive insulin therapy studies that had a target below 110 mg/dl [5,8]. The rate of severe hypoglycemia in the intensive insulin therapy ranged from 5.1 to 28.6% [5,9-13]. The rate of severe hypoglycemia was lower in our study (3.6%) compared with previous studies that adopted intensive glucose control. Moderate glucose control was safer than intensive glucose control regarding severe hypoglycemia.

There are some limitations to our study. First, this was a single-center study limited to medical ICU patients without chronic renal loss. These results do not represent all critically ill patients. Second, our study was not double-blind because safe insulin titration required the monitoring of blood glucose levels. Doctors and nurses should be aware of target glucose control levels. All patients did follow the same protocol, however, except for those following the glucose control protocol. Third, the study group was only moderately sized. Some parameters, such as clinical outcomes, did not show a significant difference due to the small sample size. Further studies are needed to examine larger sample sizes.

## Conclusion

Patients treated with MIT had significantly lower urinary nitrogen excretion and higher serum albumin and prealbumin concentrations compared with patients treated with CIT. Moderate glucose control can result in a less negative nitrogen balance in medical ICU patients. This difference was more significant in the early stages compared with the late stages of critical illness.

## Key messages

• Patients treated with MIT had significantly lower urinary nitrogen excretion and higher serum albumin and prealbumin concentrations than patients treated with CIT.

• Moderate glucose control can result in a less negative nitrogen balance; this may be related to insulin.

• The difference of nitrogen balance between MIT and CIT was more significant in the early stages compared with the late stages of critical illness in medical ICU patients.

## Abbreviations

CIT: conventional insulin therapy; IGF: insulin-like growth factor; IL: interleukin; MIT: moderate insulin therapy; TNF: tumor necrosis factor; UUN: urinary urea nitrogen.

## Competing interests

The authors declare that they have no competing interests.

## Authors' contributions

C-WH was the main contributor to the design of the study, interpretation of the data, and drafting of the manuscript. S-FS contributed to the acquisition and analysis of the data. S-LL contributed to design of the study and revision of the manuscript. K-FW contributed to statistical analysis of the data. H-HH contributed to the execution of the study. All authors read and approved the final manuscript.

## Supplementary Material

Additional file 1 **Appendix 1 showing the insulin protocol**. Click here for file
