# Supplementary material for: Moderate glucose control results in less negative nitrogen balances in medical intensive care unit patients: a randomized, controlled study
Source: Crit Care. 2012 Apr 5;16(2):R56. doi: 10.1186/cc11299 (PMC3681385; doi:10.1186/cc11299)
Supplement: Additional file 1 — Appendix 1 showing the insulin protocol. [file cc11299-S1.DOC]

**Appendix**

In the MIT group, continuous insulin infusion (50 IU of Actrapid HM) in 49.5 mL of 0.9 percent NaCl with the use of a pump was started when the blood glucose concentrations exceeded 140 mg/dL in order to maintain blood glucose concentrations between 120 and 140 mg/dL. Blood glucose levels were controlled using the neuro-fuzzy method (Table A). An antecedent insulin treatment regimen was used as a reference for the starting dose of insulin. Patients on a controlled diet, oral hypoglycemia agents, or up to 12 units/day of insulin were started with 0.5 unit/h of insulin; patients previously receiving insulin and requiring more than 12 units/day received an additional 0.5 unit/h for every 10 units exceeding 12 units/day. Blood glucose concentrations were measured every 1 to 4 h in all patients. More frequent blood glucose measurements were performed whenever the doctor or nurses considered them necessary or if there was a steep rise or fall in blood glucose or a low blood glucose level. The first row at the top of the chart in the appendix displays the range of blood glucose values measured, while the first column on the left displays the range of possible blood glucose values measured 1–4 h previously. The adjusted infusion rate is at the intersection between the perpendicular lines drawn from the present blood glucose values and the blood glucose values found 1–4 h previously.

In the CIT group, blood glucose levels were also controlled using neuro-fuzzy method (Table B). Continuous insulin infusion was delivered when the blood glucose concentrations exceeded 200 mg/dL, after which the insulin levels were adjusted to maintain blood glucose concentrations between 180 and 200 mg/dL.

If the blood glucose concentrations decreased ≤ 60mg/dL, the protocol directed the nurses to stop insulin infusion. Both moderate and severe hypoglycemia were treated by discontinuing insulin infusion and administering intravenous 50% dextrose immediately; blood glucose measurements were repeated after 30 minutes.

**Table A**

# Control of blood glucose levels in moderate insulin treatment group

Present Blood Glucose Value (mg/dL)

| Preceding Blood Glucose Value (mg/dL)  (1- 4 h before) |  | ≤60 | 61–  80 | 81–  100 | 101–  120 | 121–  140 | 141–  160 | 161–  180 | 181–  200 | 201–  220 | 221–  240 | >240 | Insulin Infusion Rate Variation (U/h) |
| --- | --- | --- | --- | --- | --- | --- | --- | --- | --- | --- | --- | --- | --- |
|  | ≤60 | -0.3 | -0.2 | 0.1 | 0.5 | 0.8 | 1.2 | 1.3 | 1.4 | 1.5 | 1.5 | 1.5 |  |
|  | 61–80 | -0.5 | -0.4 | -0.2 | 0.2 | 0.6 | 1.0 | 1.2 | 1.4 | 1.4 | 1.5 | 1.5 |  |
|  | 81–100 | -0.7 | -0.7 | -0.4 | 0.0 | 0.4 | 0.8 | 1.1 | 1.3 | 1.4 | 1.4 | 1.5 |  |
|  | 101–120 | -0.9 | -0.8 | -0.6 | -0.3 | 0.2 | 0.6 | 1.0 | 1.2 | 1.3 | 1.4 | 1.4 |  |
|  | 121–140 | -1.0 | -1.0 | -0.6 | -0.5 | 0.0 | 0.6 | 0.9 | 1.1 | 1.3 | 1.4 | 1.4 |  |
|  | 141–160 | -1.2 | -1.1 | -1.0 | -0.7 | -0.2 | 0.3 | 0.7 | 1.0 | 1.2 | 1.3 | 1.4 |  |
|  | 161–180 | -1.3 | -1.3 | -1.1 | -0.8 | -0.3 | 0.1 | 0.6 | 0.9 | 1.2 | 1.3 | 1.4 |  |
|  | 181–200 | -1.4 | -1.4 | -1.2 | -1.0 | -0.4 | -0.1 | 0.4 | 0.8 | 1.1 | 1.3 | 1.4 |  |
|  | 201–220 | -1.4 | -1.4 | -1.3 | -1.1 | -0.5 | -0.3 | 0.2 | 0.7 | 1.0 | 1.2 | 1.3 |  |
|  | 221–240 | -1.5 | -1.5 | -1.4 | -1.2 | -0.6 | -0.5 | 0.1 | 0.6 | 0.9 | 1.2 | 1.3 |  |
|  | >240 | -1.5 | -1.5 | -1.4 | -1.3 | -1.0 | -0.6 | 0.0 | 0.5 | 0.9 | 1.1 | 1.3 |  |

**Table B**

# Control of blood glucose levels in conventional insulin treatment group

Present Blood Glucose Value (mg/dL)

| Preceding Blood Glucose Value (mg/dL)  (1- 4 h before) |  | ≤120 | 121–  140 | 141–  160 | 161–  180 | 181–  200 | 201–  220 | 221–  240 | 241–  260 | 261–  280 | 281–  300 | >300 | Insulin Infusion Rate Variation (U/h) |
| --- | --- | --- | --- | --- | --- | --- | --- | --- | --- | --- | --- | --- | --- |
|  | ≤120 | -0.3 | -0.2 | 0.1 | 0.5 | 0.8 | 1.2 | 1.3 | 1.4 | 1.5 | 1.5 | 1.5 |  |
|  | 121–140 | -0.5 | -0.4 | -0.2 | 0.2 | 0.6 | 1.0 | 1.2 | 1.4 | 1.4 | 1.5 | 1.5 |  |
|  | 141–160 | -0.7 | -0.7 | -0.4 | 0.0 | 0.4 | 0.8 | 1.1 | 1.3 | 1.4 | 1.4 | 1.5 |  |
|  | 161–180 | -0.9 | -0.8 | -0.6 | -0.3 | 0.2 | 0.6 | 1.0 | 1.2 | 1.3 | 1.4 | 1.4 |  |
|  | 181–200 | -1.0 | -1.0 | -0.6 | -0.5 | 0.0 | 0.6 | 0.9 | 1.1 | 1.3 | 1.4 | 1.4 |  |
|  | 201–220 | -1.2 | -1.1 | -1.0 | -0.7 | -0.2 | 0.3 | 0.7 | 1.0 | 1.2 | 1.3 | 1.4 |  |
|  | 221–240 | -1.3 | -1.3 | -1.1 | -0.8 | -0.3 | 0.1 | 0.6 | 0.9 | 1.2 | 1.3 | 1.4 |  |
|  | 241–260 | -1.4 | -1.4 | -1.2 | -1.0 | -0.4 | -0.1 | 0.4 | 0.8 | 1.1 | 1.3 | 1.4 |  |
|  | 261–280 | -1.4 | -1.4 | -1.3 | -1.1 | -0.5 | -0.3 | 0.2 | 0.7 | 1.0 | 1.2 | 1.3 |  |
|  | 281–300 | -1.5 | -1.5 | -1.4 | -1.2 | -0.6 | -0.5 | 0.1 | 0.6 | 0.9 | 1.2 | 1.3 |  |
|  | >300 | -1.5 | -1.5 | -1.4 | -1.3 | -1.0 | -0.6 | 0.0 | 0.5 | 0.9 | 1.1 | 1.3 |  |
